# Genetic Risk Score for Coronary Heart Disease: Review

**DOI:** 10.3390/jpm10040239

**Published:** 2020-11-20

**Authors:** Sergey Semaev, Elena Shakhtshneider

**Affiliations:** 1 Institute of Internal and Preventive Medicine—Branch of Institute of Cytology and Genetics, Siberian Branch of Russian Academy of Sciences (SB RAS), Bogatkova Str. 175/1, Novosibirsk 630089, Russia; semaev@bionet.nsc.ru; 2 Federal Research Center Institute of Cytology and Genetics, SB RAS, Prospekt Lavrentyeva 10, Novosibirsk 630090, Russia

**Keywords:** genetic risk score, risk factor, myocardial infarction, coronary heart disease, cardiovascular disease

## Abstract

The present review deals with the stages of creation, methods of calculation, and the use of a genetic risk score for coronary heart disease in various populations. The concept of risk factors is generally recognized on the basis of the results of epidemiological studies in the 20th century; according to this concept, the high prevalence of diseases of the circulatory system is due to lifestyle characteristics and associated risk factors. An important and relevant task for the healthcare system is to identify the population segments most susceptible to cardiovascular diseases (CVDs). The level of individual risk of an unfavorable cardiovascular prognosis is determined by genetic factors in addition to lifestyle factors.

## 1. Introduction

Cardiovascular diseases (CVDs) play a leading role in terms of adverse effects on life expectancy and mortality globally [1]. The concept of risk factors (RFs) is generally recognized on the basis of epidemiological findings in the 20th century; according to this concept, the high prevalence of diseases of the circulatory system is due to lifestyle characteristics and associated RFs. Among the RFs of diseases of the circulatory system and myocardial infarction (MI), there are conventional (smoking, arterial hypertension, dyslipidemia, diabetes mellitus, and abdominal obesity) and unconventional, including psychosocial RFs (stress, anxiety and depression, an income level, marital status, and family conflicts). According to the results of the international INTERHEART study (conducted in 52 countries), much like hypertension and abdominal obesity, unconventional RFs are important indicators of MI risk [2]. An important and relevant task for the healthcare system is to identify the population segments most susceptible to CVDs. The level of individual risk of an unfavorable cardiovascular prognosis is determined by genetic factors in addition to lifestyle factors. The relative risk of new coronary events in patients with high genetic risk is 91% higher than that in people with low genetic risk (hazard ratio (HR) = 1.91; 95% confidence interval (CI); 1.75–2.09) [3]. It has been shown that structural changes in DNA independently affect overall mortality caused by cardiovascular events and MI [3,4,5,6,7,8,9,10,11]. The risk of an adverse outcome depends on the presence of a particular allele or genotype.

Cardiovascular risk is the likelihood of one or another adverse cardiovascular event (including death from CVD or complications) over a period of time. In clinical and research practice, several instruments are used to assess the total risk of cardiovascular pathology (Framingham, Systematic Coronary Risk Evaluation (SCORE), and Prospective Cardiovascular Munster Study (PROCAM)).

The Framingham Risk Scale was proposed according to the results of the longest prospective study (Framingham Heart Study, 1949–1984) conducted in the United States, which included 5209 people. This tool allows to assess 10-year risk of fatal and nonfatal cardiovascular complications, and this risk has 4 grades: low (risk of complications less than 10%), medium (risk greater than 10% and less than 20%), high (>20%), and very high (>30%). To calculate the total score, 5 factors are taken into account: unmodifiable (sex and age) and modifiable ones (smoking, total cholesterol, and systolic blood pressure) [12]. This risk calculator has shown good prognostic power in some cohorts similar to those in which it was designed [13], but it is known to overestimate the risk in other ethnic groups [14] and in other populations of European ethnicity where there is a lower incidence of coronary heart disease (CHD) [15,16].

The SCORE (Systematic Coronary Risk Evaluation) scale was created in Europe in 2003 on the basis of 12 cohort studies and data on 205,178 patients [17]. It predicts the possibility of an adverse outcome of CVDs by taking into account sex, age, systolic blood pressure, total cholesterol, and smoking status. This instrument allows us to calculate the risk of death from all CVDs, takes into account the multifactorial nature of the etiology of diseases, makes it possible for doctors from different countries to determine the risk, and clearly demonstrates an increase in the risk with age [18]. It has some limitations: this risk calculator is intended for people from 40 to 65 years old and does not take into consideration the level of low-density lipoprotein cholesterol (LDL-C), blood glucose, overweight, and abdominal obesity.

The PROCAM (Prospective Cardiovascular Munster Study) scale was developed from the results of a prospective study, PROCAM (Munster, Germany), which began in 1979. The study involved 21,306 people (14,799 males aged 40 to 65 years and 6507 postmenopausal females) [19]. This model is based on three unmodifiable RFs (age, a history of MI, and a hereditary history of relevant diseases) and six modifiable ones (smoking status, systolic blood pressure, LDL-C, high-density lipoprotein cholesterol, triglycerides, and the presence of diabetes mellitus). A risk less than 20% is considered low, and greater than 20% is considered high.

A family history of relevant diseases is of no small importance for assessing cardiovascular risk. Risk calculators addressing the contribution of genetic factors are under development [3,4,5,6,7,8]. The complexity of their creation is due to the population-specific prevalence of CVDs, characteristics of climatic and social living conditions, and genetic factors.

The present review deals with the stages of creation, methods of calculation, and the use of a genetic risk score (GRS) for coronary heart disease (CHD) in various populations.

## 2. History of Development of Genetic Risk Calculators

In 2005, Horne B.D. et al. described a method for calculating a GRS for predicting CHD, and this tool was based on a combination of genotyping data on single-nucleotide polymorphisms (SNPs) [20]. Three SNPs of the cholesterol metabolic pathway were evaluated for 3172 patients after coronary angiography. The GRS method revealed significant differences in the risk of CHD across GRS groups, while these SNPs when considered either separately or simultaneously as independent variables did not show a substantial influence on the risk. The potential usefulness of this method for assessing the multigenic risk of CHD was demonstrated (Table S1) [20]. The utility of the proposed genetic risk calculator was confirmed in 2007 by means of a prospective study on the risk of atherosclerosis (Atherosclerosis Risk in Communities Study, ARIC) [21]. That study showed that a GRS can improve a CHD-predictive ability as compared to a risk calculator consisting of only traditional risk factors (TRFs; Table S1) [21].

In subsequent years (2008–2015), studies had been carried out with different variations of the GRS and indicated correlations with CHD, MI, or other CVDs (Table S1) [4,5,6,7,22,23,24,25,26,27,28,29,30,31,32,33,34,35,36,37,38,39,40]. Nonetheless, several studies have not detected such an association (Table S1) [41,42,43].

To obtain estimates of the risk of CVDs in individuals by means of the GRS, the Cox proportional hazards model and logistic regression analysis have been used mainly [44,45,46,47].

Discrimination and reclassification have been the criteria for GRS quality in such studies (Table S1). Discrimination measures the ability of a prognostic method to distinguish patients with CHD from patients without CHD. Reclassification means that individuals are reassigned from one risk category to another. One of the common quantitative estimates of discrimination is the area under the receiver-operating characteristic (ROC) curve (area under the ROC curve, AUC) or the C-statistic [48,49,50]. In 2007, Pencina J. M. et al. introduced new quantitative estimates of an increase in prognostic ability toward a disease in a new method as compared to an old one with proven clinical usefulness: net reclassification improvement (NRI) and integrated discrimination improvement [51].

## 3. Modern Models of Genetic Risk Calculators (2016–2020)

Antiochos P. et al. have evaluated prognostic power of a combination of a family history of diseases and GRSs (38, 53, and 153 SNPs) for CHD cases among whites from a prospective study, CoLaus. After adjustment for cardiovascular RFs, both the family history and weighted polygenic GRSs were found to be associated with CHD, both jointly and separately, and provided additional information for predicting coronary events [52].

Tada H. et al. have tested whether the prognostic ability of a GRS for CHD would improve with an extension of GRS-27 published in 2015 by Mega J.L. et al. [7] up to a version composed of 50 SNPs and also determined whether these GRSs are independent of a family history of CHD. Those authors came to the following conclusions: (1) The addition of 23 SNPs to the previously published GRS-27 improves the assessment of CHD risk; (2) these GRSs are independent of the family history. CHD risk assessment using a GRS can be especially useful for young people [8].

Vaara S. et al. have created 3 partially overlapping GRSs (47 SNPs significantly associated with CHD in previous studies, 153 SNPs with significant or presumptive association with CHD, and 32 SNPs from the first detected CHD-associated genomic loci) and tested their prognostic ability for a relapse of acute coronary syndrome (ACS). For GRS-47, a significant association with ACS recurrence was confirmed regardless of clinical factors (HR = 1.17, 95% CI: 1.01–1.36, *p* = 0.037). For GRS-153, the association with relapses of ACS and/or mortality was not confirmed; however, an association of GRS-32 with a three-vessel lesion was found, and, for GRS-47, an inverse correlation with smoking and MI featuring ST elevation was revealed [53].

In a study by Hindieh W. et al., a family history after adjustment for sex, age, TRFs, and GRS was associated with multi-vessel lesions in CHD, as was the GRS after adjustment for TRFs including the family history. Thus, both the GRS and a family history provided important information related to the severity of CHD at a young age (≤55 years) [54].

Despite the high prevalence of cardiovascular RFs, such as hypertension, diabetes mellitus, and obesity, Afro-Caribbean mortality from CHD is lower than that of whites. Larifla L. et al. have evaluated the association of two GRSs—consisting of overlapping sets of 19 and 14 SNPs previously associated with CHD in whites—with CHD in Afro-Caribbean countries. This study indicated that the tested multilocus GRSs are strong predictors of this disease in Afro-Caribbeans. Significant differences in the allele distribution of 17 SNPs were noted between the two ethnic groups (Afro-Caribbeans and whites) [55].

### 3.1. From Tens to Tens of Thousands of SNPs

Abraham G. et al. have created a GRS of 49,310 SNPs based on a meta-analysis by the CARDIoGRAMplusC4D Consortium and tested it on five prospective population cohorts (three FINRISK and two Framingham Heart Study cohorts). For the studied GRS, an association with CHD was found in two cohorts (FINRISK: HR = 1.74, 95% CI: 1.61–1.86; Framingham: HR = 1.28, 95% CI: 1.18–1.38). Integration of the GRS with the Framingham Risk Score or ACC/AHA13 improved 10-year risk prediction, especially for people over 60. The high genomic risk was partially offset by low systolic blood pressure and cholesterol levels as well as by nonsmoking status. Those authors concluded that a GRS based on a large number of SNPs improves the risk assessment of CHD [56].

Joseph P.G. et al. have investigated the contribution of a GRS—consisting of 25 SNPs associated with CHD in Europeans and South Asians—to MI risk in various populations, with six ethnic groups as an example (Europeans, South Asians, Southeast Asians, Arabs, Hispanics, and Africans from the INTERHEART cohort). The GRS was significantly associated with MI with minimal observed heterogeneity in Europeans, South Asians, Southeast Asians, and Arabs but not in Hispanics and Africans. In the general cohort, there were only minor changes in the prognostic ability as compared to clinical factors alone [57].

Sotos-Prieto M. et al. have examined the interaction and presence or absence of synergy between the Lifestyle Cardiovascular Risk Score (LCRS) and a GRS (14 SNPs associated with MI) in the adult Hispanic/Latino population of Costa Rica. Odds ratios (ORs) for MI were 2.72 (95% CI: 2.33–3.17) per LCRS unit and 1.13 (95% CI: 1.06–1.21) per GRS unit, and there was a significant joint association for high tertiles of the GRS and LCRS as compared to low tertiles. According to those authors, improvements in a lifestyle can help prevent MI regardless of genetic predisposition [58].

Amit V. Khera et al. have estimated the extent to which a healthy lifestyle can offset an increased genetic risk of CHD. The relative risk of new coronary events in participants with high genetic risk was 91% higher relative to those with low genetic risk. Regardless of the category of genetic risk, with a healthy lifestyle (defined as the presence of at least three out of four healthy-lifestyle factors), the risk of coronary events was significantly lower than that with an unhealthy lifestyle (complete absence of healthy-lifestyle factors or the presence of only one of them). In all four samples with a total of 55,685 participants drawn from prospective cohorts of ARIC, the Women’s Genome Health Study (WGHS), the Malmö Diet and Cancer Study (MDCS), and the BioImage Study, genetic and lifestyle factors independently influenced the susceptibility to CHD. In participants with a high genetic risk, a healthy lifestyle decreased the relative risk of CHD by almost 50% as compared with an unhealthy lifestyle [3].

Widespread genetic risk variants can contribute to the heritability of early CHD. Christiansen M.K. et al. have assessed the association of a GRS (45 SNPs) with age at CHD onset and examined the relation between the GRS, familial clustering, and severity of CHD at its early onset. A higher risk shown by the GRS was registered in patients with early-onset CHD compared with late CHD and controls, but the GRS did not correlate with the stratified log-rank family score, which was employed to assess family clustering. Nevertheless, there was a significant association of the stratified log-rank family score with CHD (OR = 2.0, 95% CI: 1.4–3.0), suggesting that the analysis of a family history and TRFs are of greater clinical relevance than the GRS in question [59]. Additionally, this GRS (45 SNPs) was tested in relation to the recurrence of cardiovascular events (MI, coronary revascularization, and cardiovascular death) in patients with a confirmed diagnosis of CHD. The most pronounced effect of this GRS (for a high risk score) was detected for coronary revascularization (adjusted HR = 2.10, 95% CI: 1.08–4.07). The risks of death from cardiovascular events and of death of all causes did not change [60].

Shahid S.U. et al. have analyzed the genetic risk of CHD in individuals from Pakistan by means of a GRS consisting of 21 variants in 18 genes and determined whether this GRS is associated with blood lipid levels. The GRS was found to be associated with CHD, and the OR was statistically significantly higher in the top quintile than in the bottom quintile (2.96, 95% CI: 1.71–5.13). A strong correlation of atherogenic blood lipids with the GRS was noted too: an increase in atherogenic and a decrease in atheroprotective lipids’ levels were shown to be associated with a higher GRS result [61].

Fritz J. et al. in their study showed an association of a family CHD history and of the GRS-50 used by Tada et al. [8] with CHD (HR = 1.52, 95% CI: 1.39–1.65 and 1.53, 95% CI: 1.39–1.68, respectively). They concluded that some of the risk of CHD associated with a family history or the GRS result is mediated by elevated blood lipids (along the apoB pathway: −8.3% and 8.1%) and hypertension (8.5% and 4.2%, respectively) but not diabetes mellitus (1.5% and −0.9%, respectively). Nonetheless, most (≥80%) of the genetic effect is independent of metabolic RFs [62].

Psychological stress is an independent RF of CVDs, but the mechanism via which stress is linked with CVDs is not well studied. The purpose of a study by Svensson T. et al. was elucidation of the association between (i) a GRS (50 SNPs) (with previously proven association with CHD [8]), individual genetic variants (SNPs from the GRS that are associated with increased risk), and stress and (ii) CHD, fatal MI, nonfatal MI, or cardiovascular death. No independent association of stress with any of the endpoints and no interaction of stress with GRS were found. For the first time, it was revealed that the GRS predicting CHD (upper quartile HR = 1.72, 95% CI: 1.51–1.96) also significantly predicts fatal MI (upper-quartile HR = 1.62, 95% CI: 1.23–2.15), nonfatal MI (upper-quartile HR = 1.55; 95% CI: 1.31–1.84), and cardiovascular death (upper-quartile HR = 1.29, 95% CI: 1.08–1.53) [63].

On the basis of earlier research [7,64,65], Natarajan P. et al. have tested (in the third randomized controlled trial of a primary prevention modality (WOSCOPS)) whether statin therapy reduces relative risk of a first coronary event in individuals at high genetic risk by means of an extended GRS (57 SNPs). They also determined whether there is an association between the GRS and coronary artery calcification (CARDIA) and the severity of carotid plaques (BioImage). Among the WOSCOPS study participants with a high genetic risk, statin therapy was associated with a relative risk reduction of 44% (95% CI: 22–60; *p* < 0.001), while in all other patients, the relative risk reduction was 24% (95% CI: 8–37; *p* = 0.004), despite a similar decrease in LDL-C levels. Additionally, each increase in GRS by 1 standard deviation was associated with a 1.32-fold increase (95% CI: 1.04–1.68) in the likelihood of coronary artery calcification and a 9.7% increase (95% CI: 2.2–17.8) in the severity of a carotid plaque [66].

Paquette M. et al. have shown that a GRS containing 192 SNPs predicts CVDs in general and CHD in particular (OR = 1.82, 95% CI: 1.35–2.47 and OR = 1.76, 95% CI: 1.29–2.41, respectively, *p* < 0.001) in patients with familial hypercholesterolemia (FH). These results indicate that even in a severe monogenic disease such as FH, a GRS can improve the risk assessment of CVDs, thereby possibly facilitating a more personalized approach to treatment [67].

Although most studies are focused on identifying genetic factors of CVDs, only a few deal with a GRS to improve the prediction of recurrence in patients with established CVDs; the latter approach may be of therapeutic value in the management of these patients [7,42,53]. Accordingly, the aim of the study by Pereira A. et al. was the assessment of long-term cardiovascular mortality in individuals with CHD from Southern Europe by means of a GRS (32 SNPs). At the end of the observation period (25.8–88.1 months), the estimated survival probability was 70.8% for a high GRS value and 80.8% for a low GRS value. This work also emphasizes the relevance of genetic profiling to survival after CHD [68].

Another study was conducted by Zhao C. et al. to investigate whether a GRS can assess the risk of major adverse cardiovascular events (MACEs) in patients with established CHD. Study participants with CHD receiving statin therapy and with hypertension manifested an association between weighted GRS and MACEs. Such individuals with a medium or high risk according to the GRS also had a 2.138- and 4.048-fold higher risk of a MACE, respectively [69].

Pereira A. et al. have evaluated the utility of adding a multilocus GRS to the Framingham Risk Score during the risk assessment of CHD. Multivariate analysis showed that the GRS is an independent predictor of CHD onset (OR = 1.87, *p* < 0.0001). The tested GRS added prognostic value to TRFs in all risk subgroups [70].

One of severe subtypes of CHD is left main coronary artery disease (LMCAD), which is associated with adverse clinical outcomes [71] and has a genetic contributing factor [72,73]. Xiu Z. et al. have added three SNPs associated with an inflammatory response and initiation of CHD processes into a GRS to predict LMCAD. After adjustment for sex, age, and clinical variables related to CHD (e.g., body–mass index, smoking status, hypertension, hyperlipidemia, and diabetes mellitus), a high-risk group according to the GRS had an increased probability of LMCAD (OR = 2.78, 95% CI: 1.69–4.58, *p* = 0.02) as compared to a low-risk group [74].

### 3.2. MetaGRS

For the primary prevention of CHD, Inoye M. et al. have constructed a GRS (metaGRS) based on 1.7 million SNPs identified by a meta-analysis and assessed its potential. All three GRSs tested—(1) 46,000 SNPs from Metabochip, (2) 202 SNPs significantly associated with CHD, (3) a whole-genome GRS from the 1000 Genomes Project—turned out to be associated with CHD (HR = 1.71, 95% CI: 1.68–1.73). In an analysis of nearly 500,000 individuals from a prospective nationwide cohort study (UK Biobank), those authors evaluated a combined genomic risk metric (metaGRS) based on the summary statistics of the largest previous genome-wide studies on associations with CHD. They reported several discoveries that significantly advance the concept of applying genomic information to help stratify people at risk of CHD in general populations. MetaGRS yielded greater risk discrimination than did previously published genomic risk calculators based on selected SNPs. For instance, those authors found that metaGRS has a higher HR and positive predictive value at any given sensitivity as well as a 4-fold HR for CHD when comparing people in the upper and lower quintiles of the risk score distribution. Because these findings suggest that the higher genetic risk can at least in part be diminished by lipid-lowering and/or hypotensive therapy, people at high genetic risk may get the greatest benefit from starting these treatments early. On the other hand, the GRSs currently in use also predict the risk of CHD in people already on statin therapy for CHD, thus highlighting the need to develop new modalities to reduce the residual risk of the disease. At the same time, the genetically determined risk of CHD is mostly independent from common RFs such as blood lipids, blood pressure, and smoking. Therefore, the GRS developed in that study helps to significantly improve the stratification of people by CHD risk in various populations and shows potential usefulness of genomic screening at an early age in addition to traditional risk assessment techniques [75].

In another study, researchers analyzed four SNPs affecting coronary arteries and genotyped them in a Pakistani ethnic group. For each individual SNP, no statistically significant associations with CHD were found, in contrast to the GRS constructed from them (*p* = 1.4 × 10^−4^). Nevertheless, those authors note that a panel of SNPs included in a GRS should be designed carefully, in particular, the predisposition to the disease in different ethnic groups should be taken into consideration [76].

The vast majority of cardiovascular genomic studies have been conducted on populations of European descent. Thus, study subjects of African, Latin American, or Asian ancestry remain underrepresented in this research field. Iribarren C. et al. have investigated two GRSs (12 and 51 SNPs) previously associated with CHD in Europeans among individuals of African American, Hispanic, and East Asian descent. In a comparison of the third tertile with the first, GRS-12 showed an association with an increased risk of CHD in African Americans and Hispanics but not East Asians (HR = 1.86, 95% CI: 1.15–3.01, HR = 1.52, 95% CI: 1.02–2.25, and HR = 1.19, 95% CI: 0.77–1.83, respectively). For GRS-51, an association was identified for Hispanics and East Asians but not for African Americans (HR = 1.40, 95% CI: 0.95–2.06, HR = 1.43, 95% CI: 0.93–2.22, and HR = 1.49, 95% CI: 0.93–2.39, respectively). In a meta-analysis, both GRSs proved to be associated with the risk of CHD with a similar effect size [77].

In developing countries, the average age at the first signs of CHD (usually angina pectoris) typically exceeds 60 years [78]. Nevertheless, there is evidence that lipid streaks, which are precursors of atherosclerosis and therefore CHD, are formed in virtually all adolescents [79]. In a study by Battram T. et al., genetic variants (146 SNPs) affecting the risk of CHD in adults were reported to be associated with large changes in metabolite levels in individuals as young as 7 years of age. The identified variants are mainly related to lipid loci, and the metabolites with which they are associated are mostly related to lipoproteins. Along with further research, this knowledge may enable preventive measures such as increased monitoring of individuals at risk and possibly treatment at an earlier age, several years before the manifestation of any symptoms [80].

Liu R. et al. have devised a GRS called FDR-267 (named after the false discovery rate, FDR) from markers that were significantly associated with CHD in a meta-analysis of the UK Biobank cohort with an FDR < 5%. FDR-267 was tested on the ARIC cohort in European and African American groups. GRS FDR-267 turned out to be associated with an increase in odds ratio (OR) and HR in the European population: 1.45 (95% CI: 1.39–1.51) and 1.32 (95% CI: 1.26–1.38), respectively, which slightly improved AUC after addition to the clinical model (ΔAUC = 0.0112, *p* = 0.0002). Besides, in the European group, FDR-267 predicted CHD (C-statistic = 0.60) but showed no improvement over clinical RFs (ΔAUC = 0.0159, *p* = 0.0965). The predictive power of FDR-267 for CHD was lower in African Americans. Consequently, FDR-267 was significantly associated with CHD in the European sample, with an effect size comparable to that of the family history. FDR-267 discriminated individuals with and without CHD but did not improve the risk assessment of CHD as compared to clinical variables alone [81].

Ntalla I. et al. have tested the relation of a GRS (composed of 300 SNPs) with CHD, TRFs of CHD, and nonvascular diseases (kidney disease, migraine, and rheumatoid arthritis) that have previously proven a genetic contribution of magnitude similar to that of CHD. The GRS was found to be associated with 22 traits, including RFs, diseases secondary to CHD, and comorbid and non-CVD conditions. Sensitivity analyses were performed on individuals without CHD or with stable angina pectoris to determine the relation of the CHD-associated GRS with genetic susceptibility to these 22 traits. Hypercholesterolemia (OR = 1.27, 95% CI: 1.26–1.29) and hypertension (OR = 1.11, 95% CI: 1.10–1.12) proved to be closely associated with the GRS, indicating that this rating scale contains genetic variants predisposing to these conditions. Nevertheless, the GRS was also significantly associated with CHD cases that lacked TRFs (OR = 1.37, 95% CI: 1.30–1.44). The study also uncovered significant associations between this GRS and peripheral arterial disease (OR = 1.28, 95% CI: 1.23–1.32), abdominal aortic aneurysm (OR = 1.28; 95% CI: 1.20–1.37), and stroke (OR = 1.08, 95% CI: 1.05–1.10); these associations remained significant in a sensitivity analysis under the assumption of a general genetic predisposition. GRS was also found to correlate with heart failure (OR = 1.25, 95% CI: 1.22–1.29), atrial fibrillation (OR = 1.08, 95% CI: 1.05–1.10), and premature death (OR = 1.04, 95% CI: 1.02–1.06). These associations did not survive a sensitivity analysis, which showed that they were secondary to CHD. There was an inverse correlation between the GRS and migraine (OR = 0.94, 95% CI: 0.93–0.96). Therefore, a wide range of CVDs, including premature death, can develop sequentially or in parallel with CHD against the same genetic background [82].

Sjögren M. et al. have tested the predictive ability of the GRS (50 SNPs) developed by Tada [8] for CHD in terms of hospitalization and mortality of individuals without CHD initially. Individuals in the highest GRS quintile were found to be hospitalized 10% more often than people in the lowest quintile (incidence rate ratio = 1.10, 95% CI: 1.04–1.16, *p* = 0.001), mainly for cardiovascular reasons (incidence rate ratio = 1.31, 95% CI: 1.20–1.43, *p* = 5.17 × 10^−10^). These patients had a significantly increased risk of death from CVDs (HR = 1.44, 95% CI: 1.25–1.66, *p* = 6.56 × 10^−7^) but not the risk of death of other causes. These results indicate that a genetic predisposition to CHD allows one to predict the severity of hospitalization and mortality, especially that of cardiovascular causes, regardless of TRFs [83].

The purpose of a study by Severance L.M. et al. was to determine the optimal age for screening for coronary artery calcification by means of a GRS (associated with CHD risk in a meta-analysis [84]) in a multiethnic cohort. According to their results, this GRS is associated with a nonzero level of coronary artery calcification in the multiethnic cohort and in each ethnic group separately and can be utilized to calculate optimal age for the first test for coronary artery calcification. In addition, those authors attempted to evaluate the usefulness of the commercial 23andMe v5 chip available to ordinary consumers. Individuals from the European-American group in quintiles 3–5 of the GRS consisting of 102 SNPs featured a statistically significantly increased risk of coronary artery calcification relative to the first quintile [85].

Rincon L.M. et al. have investigated whether a GRS improves the prognosis of recurrent cardiovascular events (death, relapse, and hospitalization) in patients <55 years of age with acute MI without concomitant diabetes mellitus. These researchers also tested whether this GRS detects a more aggressive type of atherosclerosis. Compared to the general population, study participants manifested higher prevalence of risk alleles (9 of 11). A significant correlation of the GRS with recurrence of cardiovascular events was found, especially in individuals with initially elevated LDL-C levels. Compared to the low-risk GRS tertile, the multi-adjusted HRs for recurrence were 10.2 (95% CI: 1.1–100.3. *p* = 0.04) for the moderate-risk group and 20.7 (95% CI: 2.4–181.0, *p* = 0.006) for high-risk groups, where LDL-C concentration was ≥2.8 mmol/L (≥110 mg/dL). The inclusion of the GRS also bettered the C-statistic (ΔC-statistic = 0.086), NRI (30%), and integrated discrimination improvement (0.05) [86].

Earlier studies suggest that the severe monogenic disease FH is associated with an increased risk of CHD [67], but risk assessment and risk stratification remain challenging. In a study by Ellis K.L. et al., 811 patients were enrolled who visited the Lipid Disorders Clinic at Royal Perth Hospital. Pathogenic mutations were identified in 251 patients, and 560 patients were free of the pathogenic variants associated with FH. All the patients were genotyped via a GRS previously proven to be associated with CHD [8]. The GRS turned out to be associated with an increased risk of CHD in patients with identified mutations (OR = 3.3, 95% CI: 1.3–8.2, *p* = 0.009) and in patients without pathogenic variants associated with FH (OR = 1.8, 95% CI: 1.0–3.3, *p* = 0.039) after adjustment for TRFs. The GRS correlated with greater magnitude of subclinical atherosclerosis (*p* = 0.039). Patients with FH who are at high GRS risk may benefit from a more active intervention, including lifestyle changes and aggressive lipid-lowering therapy. Further evaluation of the usefulness of GRSs, including their role in the management of patients with FH in the clinic, requires a study on prospective cohorts [87].

Ohlsson M.A. et al. have studied the predictive ability of a GRS—previously used in the work of Tada H. et al. [8]—regarding possible cardiac arrest of cardiac etiology and ascertained whether it is independent of the TRFs of CHD; they also examined a combined predictive ability of the tested GRS and TRFs in terms of possible cardiac arrest. The study showed that the genetic risk of CHD in a population where this disease (and heart failure and stroke) has not been previously diagnosed is an independent RF of cardiac arrest. The statistical association was strong (HR = 1.33, 95% CI: 1.15–1.53, *p* < 0.001: cardiac arrest of cardiac etiology), and the magnitude of the risk detected by the GRS was comparable and in many cases even greater than that detected by TRFs. Given the high mortality rate of the cardiac arrest of cardiac etiology, those authors suggest that clear stratification into high-, medium-, and low-risk groups according to a GRS may help to decide on a treatment strategy, for example, prescription of lifelong statin therapy. In addition, because cardiac arrest of cardiac etiology is primarily caused by ventricular fibrillation or ventricular tachycardia, cardioverter-defibrillator implantation may be indicated for high-risk individuals as a primary prevention measure [88].

The purpose of a study by Mosley J.D. et al. was to compare CHD-prognostic power of a GRS with that of TRFs, and the genetic risk was calculated via a GRS including 6,630,149 SNPs. The GRS under study was significantly associated with a 10-year incidence of CHD in cohorts ARIC (HR = 1.24, 95% CI: 1.15–1.34) and Multi-Ethnic Study of Atherosclerosis (MESA; HR = 1.38, 95% CI: 1.21–1.58). The addition of the GRS to a risk calculator called “pooled cohort equations” did not significantly increase AUC in either cohort (ARIC: −0.001, 95% CI: −0.009 to 0.006; MESA: 0.021, 95% CI: −0.0004 to 0.043). Furthermore, there was no significant improvement in reclassification in either the ARIC sample (NRI = 0.018, 95% CI: −0.012 to 0.036) or the MESA sample (NRI = 0.001, 95% CI: −0.038 to 0.076). Those authors propose that the assessment of polygenic risk may become a motivating factor for starting a healthy lifestyle; however, they believe that there are simpler ways to promote this change [89].

Elliott J. et al. have investigated whether a GRS can improve the assessment of CHD risk as compared to the pooled cohort equations tool, which is affected by sex, race, age, blood pressure, total cholesterol, LDL-C, smoking status, and a history of diabetes mellitus. Their study involved two samples: (1) a case-control sample to optimize the predictive efficiency of CHD polygenic risk assessment on the basis of published genome-wide association studies (GWASs); (2) a prospective cohort was used to determine the predictive accuracy of the polygenic risk assessment, of the pooled cohort equations, and of a combination for incident CHD. In a cohort of 352,660 participants on whom predictive accuracy of the tested models was assessed, 6272 incident cases of CHD were registered during a median of 8 years of observation. CHD discrimination by the GRS, by the pooled cohort equations, and by their combination resulted in a C-statistic of 0.61 (95% CI: 0.60–0.62), 0.76 (95% CI: 0.75–0.77), and 0.78 (95% CI: 0.77–0.79), respectively. The change in the C-statistic between the last two models was 0.02 (95% CI: 0.01–0.03). Adding the GRS to the pooled cohort equations resulted in a 4.0% improvement in the overall NRI (95% CI: 3.1–4.9%)). The addition of the GRS to the pooled cohort equations tool resulted in a statistically significant but only modest improvement in prognostic accuracy for incident CHD and an improvement in risk stratification for only a small proportion of the subjects. Thus, the addition of genetic information to the pooled cohort equations tool requires further research before possible introduction into clinical practice [90].

Jiang J. et al. have determined whether a GRS consisting of 79 CHD-associated SNPs can predict the risk of MACEs in an ACS cohort in a prospective study of a median duration of 2 years. In the age- and sex-adjusted model, each increase in the GRS by a standard deviation was associated with a 33% increase in the risk of CVDs (HR = 1.33, 95% CI: 1.10–1.61, *p* = 0.003), with this association unabated after adjustment for TRFs. The addition of the GRS to prognostic models for seven clinical RFs and EPICOR slightly improved risk stratification for MACEs (*p* = 0.006 and *p* = 0.024, respectively). Decision curve analysis suggested that adding the GRS to the clinical factors increased CHD-predictive power when compared to each tool alone. The GRS proved to be associated with MACEs after multiple adjustment in a cohort including Chinese ACS patients [91].

### 3.3. The SNPs Most Commonly Associated with CVDs

Numerous studies carried out since the first mention of a GRS include a wide variety of SNPs (Table S2). The number of SNPs in a GRS varies from 3 [20] to >6 million [88,89]. There is a trend toward an increase in the number of SNPs included in a GRS over time. The 1378 SNPs that have been included in GRSs in various studies are presented in Table S2. To design a unified GRS, we analyzed the SNPs used in studies (from 2005 to 2020) that addressed the association of GRSs with CVDs, e.g., CHD, MI, and/or ACS, and identified the SNPs most commonly associated with heart disease, in particular, with CHD (Table 1). Additionally, Table 1 contains 25 SNPs that are most strongly associated with CHD judging by GWASs and, according to Beaney K. et al., should be included in any GRS [92].

Table 1 contains 43 single-nucleotide variants. Among them, there are genes associated with CVDs (*CDKN2B-AS1*), CHD and MI (*PHACTR1*) and genes associated with lipid metabolism (*LPA*, *LPL*, and *APOC1*), myogenesis (*VAMP5*), an immune response (*IL6R*), and angiogenesis (VEGF receptor, encoded by the *FLT1* gene, is expressed on vascular endothelial cells). The table presents both variants with low prevalence (minor allele frequency (MAF) = 0.02 (G) for rs10455872) and widespread variants with MAF > 0.3, which make up 30% of the SNPs included in the table. The majority of the single-nucleotide variants listed in the table are located in introns.

## 4. Conclusions

Thus, most authors have reported an improvement in prognostic power when a GRS is added to existing CVD risk calculators; however, some authors have not confirmed (partially or completely) the association between a GRS and CVDs [5,32,39,41,42,53,77]. This lack of associations can be explained by the need for a more thorough functional analysis of the SNPs included in GRSs. A number of authors have employed several GRSs in their work, where one GRS showed an association, and another one did not [5,53]. When a GRS is created, it is important to take into account the ethnicity of the patients included in the research [39,77]. According to many studies, the same GRS shows an association with cardiovascular events in one population and does not in another [39,77]. The problem of lacking confirmation by some authors may not necessary be solved by functional studies. A genetic association can exist even though we do not understand the function. There are other issues too: sample size, the number or type of SNPs included, statistics, ethnicity, and others. At present, there are not many published studies on the development of ethnospecific GRSs; most of the studies are carried out on white cohorts, and the results may not be applicable to other populations. Variants located in an intron that have shown an association with MI risk require further study of their functional significance in the development of CVD.

For 15 years (2005–2020) in the studies examining the association between a GRS and cardiovascular events, the methods have not undergone significant changes. Cox’s proportional hazards model and logistic regression analysis as of October 2020 remain among the most popular techniques for finding a relation between a disease and genetic factors. To measure survival, both Cox’s proportional hazards model and the Kaplan–Meier method have been used [93]. The association between a GRS and anthropometric and biochemical parameters is assessed via linear regression. ROC, AUC or the C-statistic, NRI, and integrated discrimination improvement are metrics of the quality of a GRS versus TRFs.

The evolution and improvement of the predictive ability of GRSs with the increasing number of SNPs in the model can be traced using the works of Abraham G. et al. [56] and Inoye M. et al. [75] as a vivid example. A distinctive feature of the analysis by Abraham G. et al. (as compared to several previous prospective studies examining the predictive utility of a GRS for CHD cases) is that the best predictive model was obtained with SNPs that did not necessarily achieve genome-wide or even statistical significance in previous GWASs. A GRS surpassed other models that were smaller in terms of the number of included SNPs and showed greater promise for predicting CHD between the upper and lower GRS quintiles than did the study by Tada H. et al., which tested a 50-SNP GRS on Scandinavians [8] (GRS50 HR¼ 1.92 versus GRS49K HR¼ 4.51). According to Inoye M. et al. [75], metaGRS achieved greater risk discrimination than previously published estimates of genomic risk: those authors found that metaGRS had greater HR and positive predictive value at any given sensitivity as well as 4-fold HRs for CHD when comparing people in the upper and lower quintiles of the distribution of risk estimates. It was also demonstrated that the predictive power of metaGRS was substantially independent from established CHD RFs, implying that the genetic information complements (rather than replaces) common RFs. Finally, those authors found that metaGRS identifies people at high risk of premature CHD and those who are unlikely to ever reach the lifetime risk levels that necessitate an intervention.

Although applied medical research will be necessary to properly assess the clinical utility of GRSs for CHD, areas of potential clinical application can already be foreseen. For instance, genotyping across large parts of the genome requires a one-time cost of approximately USD 50 in 2018 prices [75] and can be used to calculate updated estimates of the genomic risk of CHD as more convincing data on this association become available. Indeed, genome-wide genotyping array data can be used to calculate a GRS for a wide range of common diseases. To compute a genomic risk for individuals, simple algorithms can employ information from such datasets and from large reference groups out of similar populations such as the UK Biobank [75].

Designing a GRS for the assessment of cardiovascular risk is an important task for modern cardiology. Given that the risk of CVDs as assessed by GRSs is preventable or at least can be partially reduced (for example, by treatment with lipid-lowering medication), early diagnosis may improve the quality and duration of life of the patients and should reduce economic costs.

## Figures and Tables

**Table 1:** Single-nucleotide polymorphisms (SNPs) associated with cardiovascular diseases (CVDs) according to research data from 2005 to 2020.

SNV	Chromosome (GRCh38)	Gene	Position in the Gene	Risk Allele *	MAF	References
rs646776	1:109275908	*CELSR2*	500 bp downstream of TSS	T	0.24 (C)	[4,5,7,8,26,27,30,31,33,34,35,39,42,43,54,57,58,61,62,63,83,88,91]
rs602633 ^a^	1:109278889	*-*	-	G	0.35 (T)	[33,37,40,52,53,66,77,80,92]
rs4845625	1:154449591	*IL6R*	Intron	T	0.44 (T)	[3,8,33,37,38,40,52,53,59,60,63,66,77,80,83,87,88,91]
rs4846525 ^a^	1:216547016	*ESRRG*	Intron	-	0.26 (T)	[92]
rs17464857 ^a^	1:222589367	*TAF1A, TAF1A-AS1*	*TAF1A*: intron, *TAF1A-AS1*: 2 kbp upstream	C/T	0.08 (G)	[38,66,77,82,92]
rs17465637	1:222650187	*MIA3*	Intron	C	0.50 (A)	[3,4,5,7,8,24,27,29,31,33,34,35,39,40,42,43,53,54,55,57,59,60,61,62,63,68,70,83,86,87,88,91]
rs11206510 ^a^	1:55030366	*-*	-	A/C	0.10 (C)	[3,4,5,7,8,27,28,30,31,33,34,35,37,38,40,41,42,43,52,53,54,57,59,60,62,63,66,67,75,77,87,88,91,92]
rs17114036 ^a^	1:56497149	*PLPP3*	Intron	A	0.09 (G)	[3,5,7,8,33,34,37,38,40,42,43,52,53,54,57,62,63,66,77,83,87,88,91,92]
rs2252641	2:145043894	*TEX41*	Intron	C	0.31 (T)	[3,8,37,38,40,52,53,59,60,62,63,66,77,80,83,87,88,91]
rs6725887	2:202881162	*WDR12*	Intron	C/T	0.05 (C)	[3,4,5,6,7,8,24,27,29,30,31,33,34,35,38,41,42,43,54,57,59,60,62,63,66,83,86,88,91]
rs515135	2:21063185	*-*	-	C/T	0.25 (T)	[3,5,8,33,37,38,40,43,52,53,59,60,62,63,66,77,80,83,87,88,91]
rs1561198 ^a^	2:85582866	*VAMP5*	2 kbp upstream	T	0.49 (T)	[3,33,37,38,40,52,53,59,60,66,77,87,92]
rs9818870	3:138403280	*MRAS*	3′UTR	T	0.09 (T)	[3,4,5,6,7,8,24,29,31,33,38,39,41,42,43,54,55,58,59,60,61,62,63,66,77,82,83,86,87,88,91]
rs7692387 ^a^	4:155714157	*GUCY1A1*	Intron	G	0.16 (A)	[3,8,33,37,38,40,52,53,59,60,62,63,66,77,80,83,87,88,91,92]
rs273909	5:132331660	*SLC22A4, MIR3936HG*	*MIR3936HG*: intron, *SLC22A4*: intron	G	0.09 (G)	[3,8,33,37,38,40,52,53,59,60,62,63,66,77,80,82,83,87,88,91]
rs9369640 ^a^	6:12901209	*PHACTR1*	Intron	A	0.36 (C)	[33,66,92]
rs12526453	6:12927312	*PHACTR1*	Intron	C/A	0.17 (G)	[3,5,6,8,24,27,29,30,31,34,37,38,40,41,42,43,52,53,54,59,60,62,63,68,77,83,86,87,88]
rs12190287	6:133893387	*TCF21*	3′UTR	C	0.34 (G)	[3,5,7,8,33,37,38,40,42,52,53,54,59,60,62,63,66,68,70,77,83,87,88,91]
rs2048327	6:160442500	*SLC22A3*	Intron	A/C	0.29 (C)	[3,7,8,30,33,37,40,42,43,52,53,58,62,63,66,77,83,87,88,91]
rs3798220	6:160540105	*LPA*	Missense mutation	C	0.05 (C)	[3,4,5,7,8,34,38,39,42,43,54,55,57,59,60,61,62,63,66,77,83,87,88]
rs10455872	6:160589086	*LPA*	Intron	G	0.02 (G)	[3,7,8,29,39,55,61,62,77,81,83,85,86,87,88]
rs4252120 ^a^	6:160722576	*PLG*	Intron	T	0.14 (C)	[3,8,33,37,38,40,52,53,59,60,62,63,66,77,83,87,88,91,92]
rs12205331 ^a^	6:34930678	*ANKS1A*	Intron	C	0.08 (T)	[37,40,53,66,77,92]
rs10947789	6:39207146	*KCNK5*	Intron	T	0.17 (C)	[3,8,33,37,38,40,52,53,59,60,63,66,77,80,83,87,88,91]
rs11556924 ^a^	7:130023656	*ZC3HC1*	Missense mutation	C	0.16 (T)	[3,5,7,8,33,34,37,38,40,42,43,52,53,54,57,59,60,62,63,66,68,70,75,80,81,82,83,85,87,88,91,92]
rs2954029 ^a^	8:125478730	*-*	-	A	0.41 (T)	[3,5,8,32,33,37,38,40,41,43,52,53,59,60,62,63,66,77,81,82,83,85,87,88,92]
rs264 ^a^	8:19955669	*LPL*	Intron	G	0.16 (A)	[33,37,38,40,52,53,59,60,66,77,91,92]
rs3217992 ^a^	9:22003224	*CDKN2B, CDKN2B-AS1*	*CDKN2B-AS1*: intron, *CDKN2B*: 3′UTR	T	0.35 (T)	[3,8,33,37,40,52,53,62,63,66,77,82,83,87,88,92]
rs1333049 ^a^	9:22125504	*CDKN2B-AS1*	Intron	C	0.42 (C)	[23,24,27,29,30,33,35,37,38,40,41,43,52,53,58,59,60,66,68,70,77,86,92]
rs12413409 ^a^	10:102959339	*CNNM2*	Intron	G	0.16 (A)	[3,5,7,8,33,34,36,38,42,43,54,57,59,60,62,63,66,77,83,87,88,91,92]
rs501120 ^a^	10:44258419	*-*	-	T/C	0.33 (C)	[3,6,23,26,28,30,33,37,38,40,52,53,58,59,60,66,77,86,87,91,92]
rs1746048	10:44280376	*-*	-	C	0.34 (T)	[4,5,6,7,8,24,27,29,31,34,35,39,41,42,43,54,55,57,58,61,62,63,83,88]
rs964184	11:116778201	*ZPR1*	3′UTR	G	0.22 (G)	[3,5,7,8,32,33,34,41,42,43,52,54,57,59,60,62,63,68,70,81,82,83,85,87,88,91]
rs3184504 ^a^	12:111446804	*SH2B3*	Missense mutation	T/C	0.15 (T)	[3,4,5,7,8,30,31,33,34,37,38,40,42,52,53,54,59,60,62,63,66,77,83,87,88,91,92]
rs4773144 ^a^	13:110308365	*COL4A1, COL4A2*	*COL4A2*: intron, *COL4A1*: 2 kbp upstream	G	0.40 (G)	[3,7,8,33,34,37,38,40,42,43,52,53,54,57,59,60,62,63,66,83,87,88,91,92]
rs9515203 ^a^	13:110397276	*COL4A2*	Intron	T	0.22 (C)	[3,8,33,37,40,52,53,62,63,66,67,75,77,80,82,83,87,88,92]
rs9319428 ^a^	13:28399484	*FLT1*	Intron	A	0.33 (A)	[3,8,33,37,38,40,52,53,59,60,62,63,66,77,80,83,88,91,92]
rs2895811	14:99667605	*HHIPL1*	Intron	C	0.32 (C)	[3,5,7,8,33,34,37,38,40,42,43,52,53,54,57,59,60,62,63,66,83,88,91]
rs7173743 ^a^	15:78849442	*MORF4L1*	Intron	T	0.47 (C)	[3,8,33,37,38,40,52,53,62,63,66,77,80,83,87,88,91,92]
rs12936587 ^a^	17:17640408	*-*		G	0.27 (A)	[3,5,7,8,33,34,37,38,40,42,43,52,53,54,57,59,60,62,63,66,77,80,83,87,88,91,92]
rs1122608 ^a^	19:11052925	*SMARCA4*	Intron	G	0.14 (T)	[3,4,5,7,8,27,30,31,33,34,35,37,38,40,41,42,43,52,53,54,57,59,60,62,63,66,77,83,87,88,91,92]
rs445925 ^a^	19:44912383	*APOC1*	2 kbp upstream	G	0.15 (A)	[33,37,40,53,66,77,92]
rs9982601	21:34226827	*-*	-	T/C	0.11 (T)	[3,4,5,6,7,8,27,29,30,31,33,34,35,37,38,40,41,42,52,53,54,57,59,60,62,63,66,77,83,86,88,91]

SNV: single-nucleotide variant. * These risk alleles can differ among (or be absent in) some studies. ^a^ These SNPs are in the top 25 CHD risk loci according to GWAS data [92].

## Supplementary Materials

The following are available online at https://www.mdpi.com/2075-4426/10/4/239/s1, Table S1: Studies on genetic risk calculators, 2005–2015, Table S2: SNPs included in genetic risk scores since 2005.

## Abbreviations

ACS	acute coronary syndrome
ARIC	Atherosclerosis Risk in Communities Study
AUC	area under the ROC curve
CHD	coronary heart disease
CI	confidence interval
CVD	cardiovascular disease
FDR	false discovery rate
FH	familial hypercholesterolemia
GRS	Genetic Risk Score
GWAS	genome-wide association study
HR	hazard ratio
LDL-C	low-density lipoprotein cholesterol
LCRS	Lifestyle Cardiovascular Risk Score
LMCAD	left main coronary artery disease
MACE	major adverse cardiovascular event
MAF	minor allele frequency
MESA	Multi-Ethnic Study of Atherosclerosis
MI	myocardial infarction
NRI	net reclassification improvement
OR	odds ratio
PROCAM	Prospective Cardiovascular Munster Study
RF	risk factor
ROC	receiver-operating characteristic
SCORE	Systematic Coronary Risk Evaluation
SNP	single-nucleotide polymorphism
TRF	traditional risk factor
