# Supplementary material for: Genetic Risk Score for Coronary Heart Disease: Review
Source: J Pers Med. 2020 Nov 20;10(4):239. doi: 10.3390/jpm10040239 (PMC7712936; doi:10.3390/jpm10040239)
Supplement: Supplementary file 1 [file jpm-10-00239-s001.zip › supplementary/Table S1.docx]

Table S1. Studies on genetic risk calculators, 2005–2015.

| **Year** | **Pathology** | | | | | | **Study design** | | | **Population** | | | | | **Cohort** | | | | **Sample size** | | | | | **Number of SNPs in GRS** | | **Methods** | | **Results** | | | | | **Conclusion** |
| --- | --- | --- | --- | --- | --- | --- | --- | --- | --- | --- | --- | --- | --- | --- | --- | --- | --- | --- | --- | --- | --- | --- | --- | --- | --- | --- | --- | --- | --- | --- | --- | --- | --- |
| 2005 | IHD | | | | | | Retrospective cohort analysis | | | Patients of predominantly British / Northern European descent were chosen from a population genetically similar to the white population of the United States | | | | | - | | | | 3172 patients after coronary angiography | | | | | 3 | | Linear regression: SNPs associated with an intermediate phenotype. Logistic regression: association of GRS with IHD | | HDL-C level is significantly associated with genes *CETP* (B2B2 (GG): p < 0.001; B1B2 (AG): p = 0.006), *ABCA1* (AA: p = 0.014), and *HL* (TT: p = 0.033). No genotype was associated with LDL-C or TG levels. Reduced risk for the third group of patients compared to the fourth (OR = 0.70, 95% CI: 0.55-0.88, p = 0.003), as well as after adjustment for age, sex, and 6 TRFs (OR = 0.73, 95% CI: 0.56– 0.96, p = 0.02). | | | | | A method for calculating a genetic risk score (GRS) is described [Horne B.D. et al. 2005] |
| 2007 | IHD | | | | | | Prospective cohort analysis | | | whites | | | | | ARIC | | | | 8361 | | | | | 10 for whites, 11 for blacks, among them one common (rs20455 in *KIF6*) | | Cox proportional hazards model containing TRFs. AUC | | Each GRS improves the IHD prediction for the respective race, compared to using TRFs alone (white race: HR = 1.10, 95% CI: 1.06-1.14; black race: HR = 1.20, 95% CI: 1.11-1.29; each tertile defined via a traditional scale: p < 0.02). AUC for each race was increased (whites: Δ = 0.002, blacks: Δ = 0.011). | | | | | Studied GRSs are associated with IHD in each race [Morrison A.C. et al. 2007] |
|  |  |  |  |  |  |  |  |  |  | blacks | | | | | ARIC | | | | 2624 | | | | |  |  |  |  |  |  |  |  |  |  |
| 2008 | CVDs (first MI, IS, or death from IHD) | | | | | | Prospective cohort analysis | | | - | | | | | Malmö Diet and Cancer Study (MDCS) | | | | 4232 (average over 10.6 years: 131 cases of MI, 96 cases of IS and 11 deaths from IHD) | | | | | 9 | | Cox proportional hazards model. ROC and C-statistic. IDI | | Taking into account the levels of lipids and RFs, HR showed that GRS is associated with CVDs: 1.15 (95% CI: 1.07-1.24, p = 3 × 10^-4^). There was an improvement in the classification in terms of the score for the genotype (p = 0.01), and the IDI also had a higher value (p = 0.02). C-statistic was not improved: 0.80 | | | | | The tested GRS is associated with CVDs (Kathiresan S. et al. 2008) |
| 2010 | CVDs (MI, stroke, arterial revascularization, or cardiovascular death) | | | | | | Prospective cohort analysis | | | whites | | | | | Women’s Genome Health Study | | | | 19,313, among which 777 cardiovascular events occurred on average over 12.3 years (199 MI, 203 strokes, 63 CVD deaths, and 312 revascularizations) | | | | | 101 associated with CVDs and intermediate phenotype. 12 associated with CVDs | | Cox proportional hazards model. Base models: age plus ATP III and Reynolds covariates. NRI | | Age-adjusted HR showed a relationship between two GRSs and CVDs: GRS 101: 1.015 (95% CI: 1.00-1.03; p = 0.006), GRS 12: 1.05 (95% CI: 1.01-1.09; p = 0.014), unlike ATP III and Reynolds covariates. However, there was no improvement in discrimination and reclassification. Nonetheless, family history analysis without GRS was also associated with CVDs in multivariate models (HR = 1.67, 95% CI: 1.39-1.03; p < 0.001 and HR = 1.57, 95% CI: 1.31-1.89; p < 0.001) after adjustment for age and ATP III, respectively, and also showed an improvement in discrimination 0.709 (p = 0.013) and reclassification 3.1 (p = 0.02) after adjustment for age. | | | | | The tested GRSs are not associated with CVDs (Paynter N.P. et al. 2010) |
| 2010 | IHD | | | | | | Case-control | | | Puerto Ricans | | | | | Boston Puerto Rican Health Study | | | | 197 patients with IHD, 524 without IHD | | | | | 11 | | Genetic Predisposition Scale (GPS): 1 point: homozygote for the high-risk allele, 0.5 points: heterozygote, 0 points: homozygote for the low-risk allele. | | In 47% of patients with IHD: GPS > 5 (OR = 2.99, 95% CI: 1.76-5.09; p < 0.001) and in 66% of patients without IHD: GPS ≤ 5 (p = 0.001). The combined presence of GPS> 5 and TRFs was associated with a higher risk of IHD. | | | | | The tested GRS is associated with IHD (Junyent M. et al. 2010) |
| 2010 | IHD | | | | | | Case-control | | | - | | | | | Wellcome Trust Case Control Consortium (WTCCC) | | | | 1988 IHD patients, 5380 controls | | | | | 9, associated with IHD and unrelated to TRFs | | GRS: a patient may have 0-18 risk alleles (0, 1, or 2 risk alleles for one SNP). Logistic regression analysis | | As the number of risk alleles increases, the risk of IHD increases. OR with an increase by 1 risk allele: 1.18 (95% CI: 1.15-1.22, p = 2 × 10^-16^) | | | | | An association was found between the number of risk alleles and IHD (Lluis-Ganella C. et al. 2010) |
| 2010 | IHD | | | | | | Case-control | | | - | | | | | Intermountain Healthcare’s Angiographic Registry and DNA Bank | | | | 1918 patients with early IHD, 1032 controls | | | | | 5 associated with IHD or IHD risk indicators | | Logistic regression analysis | | OR showed an association of GRS with IHD: 1.24 (95% CI: 1.16-1.33, p = 8.2 × 10^-11^). When the first and fourth quartiles were compared, OR = 2.03 (95% CI: 1.53-2.70). The addition of GRS significantly improved net reclassification (NRI = 0.016, p < 0.0001). However, the addition of GRS did not significantly affect AUC. | | | | | The tested GRS is associated with IHD (Anderson J.L. et al. 2010) |
| 2010 | IHD (MI, UAP, coronary revascularization, or death from IHD) | | | | | | Prospective cohort analysis | | | Finland, Sweden | | | | | FINRISK 1992, 1997, и 2002, Health 2000, MDCCC | | | | 30725 (1264 participants had a first IHD event during a median 10.7 years’ follow-up) | | | | | 13 associated with MI and IHD | | Cox proportional hazards model and logistic regression analysis. ROC, AUC. NRI and IDI | | OR when the highest and lowest quintile groups were compared: for IHD: 1.63 (95% CI: 1.24-2.15, p = 4.8 × 10^–5^), for CVDs: 1.30 (95% CI: 1.15-1.47, p = 2.6 × 10^–^⁸), and for MI: 1.56 (95% CI: 1.38-1.76, p = 1.2 × 10^–^¹⁵). AUC: Δ = 0.001 (p = 0.19), 0.000 (p = 0.48), 0.001 (p = 0.35) for IHD, CVDs, and MI, respectively. IDI values: for IHD: 0.004, p = 0.0006, for CVDs: 0.004, p = 0.0004, and for MI: 0.003, p = 0.03. NRI was not significant for IHD (2.2%, p = 0.182) but significant for intermediate phenotype (9.7%, p = 3 × 10^–^⁶). | | | | | The tested GRS is associated with IHD, CVDs, and MI (Ripatti S. et al. 2010) |
|  |  |  |  |  |  |  | Case-control | | |  |  |  |  |  | FINRISK 1992, 1997, and 2002, Health 2000, MDC-CC, MPP, и COROGENE | | | | 3829 patients with IHD, 48897 controls | | | | |  |  |  |  |  |  |  |  |  |  |
| 2011 | Nonfatal acute MI | | | | | | Case-control | | | Spanish-speaking population of Costa Rica | | | | | - | | | | 1,989 patients, 2096 controls | | | | | 3 associated with IHD and/or MI in white, African, and Asian populations from the HAPMAP project | | Logistic regression analysis. ROC and AUC | | The OR for MI per unit of genetic risk was 1.18 (95% CI: 1.11-1.25; p = 4.83 × 10^-8^). When GRS was added to the model including clinical predictors, the change in AUC was small (Δ = 0.01; without GRS: 0.67, 95% CI: 0.65-0.69, and with GRS: 0.68, 95% CI: 0.66-0.70) but statistically significant (p = 0.02). | | | | | The tested GRS involving SNPs associated with Europeans is associated with IHD in Hispanics (Qi L. et al., 2011) |
| 2012 | IHD, other CVDs, e.g., high calcification of coronary arteries | | | | | | Prospective cohort analysis | | | Framingham population (USA) | | | | | Framingham Offspring и the Framingham Third Generation | | | | 3014 (539 of CVD cases and 182 severe IHD cases were identified) | | | | | 13 taken from GWASs for IHD and MI, 102 based on GWASs for TRFs of IHD with 13 SNPs included, 29 (13 + 16 added from GWASs for IHD and MI) | | Cox proportional hazards model, logistic regression analysis, NRI, IDI | | With adjustment for RFs of CVDs and for a family history, only GRS 13 showed an association with severe IHD (HR = 1.07, 95% CI: 1.00-1.15; p = 0.04), CVDs (HR = 1.05, 95% CI: 1.00-1.09; p = 0.03), and high coronary artery calcification (HR = 1.18, 95% CI: 1.11-1.26; p = 3.4 × 10^-7^). GRS did not improve discrimination for either IHD or CVDs but only slightly enhanced reclassification. However, an improvement in discrimination and reclassification was observed at high levels of coronary calcification. | | | | | GRS-13 is an independent predictor of cardiovascular events (Thanassoulis G. et al. 2012) |
| 2012 | IHD or stroke | | | | | | Case-control | | | Greece population | | | | | European Prospective Investigation into Cancer and nutrition (EPIC) | | | | 814 cases (494 IHD cases, 320 stroke cases), 1345 controls | | | | | 9 from GWAS, are associated with MI and IHD in European populations | | Logistic regression analysis adjusted for sex and age | | GRS is associated with IHD (OR = 1.21; 95% CI: 1.09-1.35; p = 0.0004). The risk of IHD according to GRS is 1.74-fold higher in the top quintile than in the bottom one (95% CI: 1.25-2.43; p = 0.0004). The risk of stroke was also 1.36-fold higher, but there were no statistically significant differences between the top and bottom quintiles (95% CI: 0.90-2.06; p = 0.188). | | | | | The tested GRS is associated with IHD (Yiannakouris N. et al. 2012) |
| 2012 | IHD | | | | | | Case-control | | | Han population in China | | | | | - | | | | 1007 cases, 889 controls | | | | | 8 associated with IHD and TRFs | | Logistic regression analysis (cGRS; wGRS), ROC, AUC | | The risk of IHD was 2.43-fold (cGRS) and 2.47-fold (wGRS) higher in the upper quintile than in the lower quintile (95% CI: 1.84-3.19; p < 0.001 and 95% CI: 1.88-3.25; p < 0.001, respectively). There was an increase in AUC at cGRS of 0.686 (Δ = 0.018, 95% CI: 0.685-0.687) and at wGRS of 0.690 (Δ = 0.022, 95% CI: 0.689-0.691). | | | | | The tested GRS is associated with IHD (Lv X. et al. 2012) |
| 2012 | IHD | | | | | | Prospective cohort analysis | | | | Population of Girona Province (North-East Spain) | | | | REGICOR (Registre Gironí del Cor) | | | | 2351 (107 events of IHD on average during 9.75 years) | | | | 8 associated with IHD but not with TRFs | | | Cox proportional hazards model adjusted for TRFs; C-statistic, NRI, and IDI | | GRS is linearly associated with IHD in both cohorts (REGICOR: p = 0.001, Framingham: p = 0.016). The addition of GRS improved the ability to predict IHD for Framingham (C-statistic: 72.81 vs. 72.37, p = 0.042) but not for REGICOR (78.35 vs. 78.33, p = 0.806). The improvement in reclassification was greater in the group with an intermediate phenotype (NRI: 17.44, 95% CI: 8.04-26.83; IDI: 0.29, 95% CI: 0.01-0.56). | | | | | The tested GRS is associated with IHD (Lluis-Ganella C. et al. 2012) |
|  |  |  |  |  |  |  |  |  |  |  | Framingham population (USA) | | | | Framingham Heart Study | | | | 3537 (429 events of IHD on average during 13.32 years) | | | |  |  |  |  |  |  |  |  |  |  |  |
| 2012 | IHD | | | | | | Prospective cohort analysis and case-control | | | | whites | | | | MORGAM project | | | | 4818 males (1736 cases over 18 years) | | | | 11 + 2 haplotypes (4 SNPs from the LPA locus), 15, 8 | | | Cox proportional hazards model, IDI, NRI, C-statistic | | GRS1 and GRS2 improved NRI as compared to Framingham score (GRS1: 7.5%, p = 0.017; GRS2: 6.5%, p = 0.044). However, discrimination was improved only by GRS2 (improvement in C-statistic by 1.11%, p = 0.048). The net reclassification for men aged 50-59 was also improved by 13.8% (GRS1) and 12.5% (GRS2). | | | | | The tested GRSs are associated with IHD (Hughes M.F. et al. 2012) |
| 2012 | IHD | | | | | | Prospective cohort analysis | | | | non-Hispanic whites | | | | ARIC, Rotterdam и Framingham Offspring Studies | | | | 8542 (13.0% of cases), 2068 (13.1% of cases), and 2339 (9.2% of cases) | | | | 13 [taken from: 1) the National Human Genome Research Institute database on the basis of phenotype/ trait; 2) from published GRSs for IHD] | | | Cox proportional hazards model including TRFs. AUC, NRI, IDI | | GRS is associated with the risk of IHD (HR = 1.10, 95% CI: 1.07-1.13). A significant improvement in discrimination and reclassification for ARIC (AUC: Δ = 0.007, 95% CI: 0.004-0.013; NRI = 6.3%). However, for Rotterdam (HR = 1.08; 95% CI: 1.02–1.14) and Framingham Offspring (HR = 1.12; 95% CI: 1.10–1.14), there was no substantial improvement in AUC and NRI. | | | | | The tested GRS is associated with IHD in the ARIC cohort (Brautbar A. et al. 2012) |
| 2012 | MI or IHD | | | | | | Prospective cohort analysis and case-control | | | | whites | | | | Cleveland Clinic GeneBank | | | | 2702 (1154 cases of IHD, 495 controls) | | | | 11 related to MI/IHD according to GWAS and publications prior to June 2009 | | | Logistic regression analysis, Cox proportional hazards model, ROC, C-statistic | | With a history of MI: the risk of MI in the Emory cohort was higher in the upper quintile compared to the lower one (OR = 1.81, 95% CI: 1.21-2.70), with the effect size being greater with younger age compared to the older control (OR = 1.90, 95% CI: 1.24-2.92 for MI at age <60 years; OR = 2.37, 95% CI: 1.44-3.91 for MI at age <50 years). Significant increase in C-statistic (<60 years: Δ = 0.013, p = 0.03; <50 years: Δ = 0.017, p = 0.04), similarly for AUC = 0.027, 0.030, 0.036 for MI at age <70, <60, and <50 years respectively. In the Cleveland Clinic cohort, there was an increased risk in the top quintile compared to the bottom one (HR = 1.08, 95% CI: 0.57-1.99). Incident MIs: top to bottom quintile ratio in the presence of MI: HR = 1.08 (95% CI: 0.57-1.99), MI/death: HR = 0.68 (95% CI: 0.49-0.94). | | | | | GRS is associated with a history of MI. Limited predictive power of the GRS for secondary risk in already established IHD (Patel R.S. et al. 2012) |
|  |  |  |  |  |  |  |  |  |  |  |  |  |  |  | Emory Cardiovascular Biobank | | | | 2597 (101 cases of MI and 358 the composite endpoint of death or MI during 2.5 years) | | | |  |  |  |  |  |  |  |  |  |  |  |
| 2013 | | | | MI or IHD | | | N/A | | | - | | | | | Erasmus Rucphen Family Study (EFR) | | | | 2269 | | | | 52 associated with TC, 37 with LDL-C, 47 with HDL-C, 32 with TGs | | | Linear regression model, Cox proportional hazards model, AUC | | An association of two GRS (which included SNPs with a previously found association with TC or LDL-C) with MI and IHD was detected (GRS-TC with MI HR = 1.12, 95% CI: 1.03-1.22, p = 0.012 and with IHD HR = 1.10, 95% CI: 1.04–1.17, p = 7.02 × 10^–4^; GRS-HDL-C with MI HR = 1.12, 95% CI: 1.03-1.23, p = 0.011, and with IHD HR = 1.10, 95% CI: 1.04-1.17, p = 7.00 × 10^–4^). There were no statistically significant results on MI and IHD for the GRSs associated with HDL-C and TGs. No improvement in AUC was observed for any of the GRSs. | | | | GRSs correlating with TC or LDL-C are associated with MI and IHD (Isaacs A. et al. 2013) | |
|  |  |  |  |  |  |  |  |  |  | - | | | | | Rotterdam Study (RS) | | | | 8130: 398 cases of MI and 924 cases of IHD | | | |  |  |  |  |  |  |  |  |  |  |  |
| 2013 | | | | IHD | | | Prospective cohort analysis | | | Sweden population | | | | | SATSA, OCTO-Twin, GENDER, HARMONY, TwinGene, Uppsala Longitudinal Study of Adult Men (ULSAM) | | | | 10612 (781 IHD events over 3.6-5.8 years) | | | | 395: general, 46: IHD-specific, and 6 feature-specific GRS for each IHD RF (BMI: 37, HDL-C: 47, SBP: 35, TC: 34 , smoking: 7, T2DM: 40, Framingham Heart Study (FHS) GRS: 180) | | | Cox proportional hazards model, C-statistic, NRI, linear/logistic regression analysis | | General and IHD-specific GRSs are associated with IHD (p = 2 × 10^-5^ and p = 4 × 10^-6^, respectively). The ratio of the last quintile to the first: total MGRS: HR = 1.54, 95% CI: 1.25-1.95; IHD-specific: HR = 1.52, 95% CI: 1.24-1.87. Increased reclassification for total MGRS: NRI = 4.2, 95% CI: 1.2-7.1, but slight improvement in discrimination: C-statistic = 0.002. For IHD-specific GRS, both reclassification (NRI = 4.9, 95% CI: 1.1-8.7) and discrimination (C-statistic = 0.004) were better. | | | | Total and IHD-specific GRSs are associated with IHD (Ganna A. et al. 2013) | |
| 2013 | | | | CVDs, e.g., IHD or ACS | | | Prospective cohort analysis | | | Finland population | | | | | FINRISK 1992, FINRISK 1997, FINRISK 2002, Health 2000 | | | | 24124 (1093 IHD events, 1552 CVD events, and 731 ACS events over 12 years) | | | | 28, associated with IHD and MI | | | Cox proportional hazards model adjusted for TRFs. C-statistic, NRI, IDI | | GRS is associated with all clinical endpoints (IHD: HR = 1.27, 95% CI: 1.20-1.35, p = 1.2 × 10^-14^; ACS: HR = 1.27, 95% CI: 1.18-1.37, p = 3.1 × 10^-10^; CVDs: HR = 1.18, 95% CI: 1.12-1.24, p = 3.2 × 10^-10^). GRS improved discrimination for all endpoints by 0.3–0.5% (p = 0.0002 for IHD, p = 0.0004 for CVDs, and p = 0.001 for ACS). The addition of GRS to the model with TRFs and a family history resulted in an overall NRI of 5% (p = 0.01). | | | | GRS is associated with CVDs, IHD, and ACS (Tikkanen E. et al. 2013) | |
| 2014 | | | | IHD | | | Case-control | | | Greece population | | | | | European Prospective Investigation into Cancer and nutrition (EPIC) | | | | 477 IHD patients, 1271 controls | | | | 9 associated with IHD and MI | | | Logistic regression analysis | | Combined presence of higher risk according to GRS and TRFs was associated with an increased risk of IHD as compared with combined presence of lower risk according to GRS and TRFs. For smoking: OR = 1.7, 95% CI: 1.2-2.4; for hypertension: OR = 2.7, 95% CI: 1.9-3.8; for T2DM: OR = 4.1, 95% CI: 2.8-6.1; for lower physical activity: OR = 1.9, 95% CI: 1.4-2.5; for high body-mass index: OR = 2.0, 95% CI: 1.3-3.2; for poor adherence to the Mediterranean diet: OR = 1.5, 95% CI: 1.1-2.1. | | | | The combined presence of TRFs and a high GRS result is more strongly association with IHD (Yinnakouris N. et al. 2014) | |
| 2014 | | | | IHD | | | Case-control | | | Population of Hubei Province, China | | | | - | | | | 1038 IHD cases of which 379 were MI cases; 1083 controls | | | | 10 associated with MI/IHD either in a European population or in the Han population | | | | Logistic regression analysis including TRFs. ROC and AUC, NRI and IDI | | | The second and third tertile showed an increased risk of IHD as compared to the first one after adjustment for TRFs (OR = 1.32, 95% CI: 1.02-1.73, p = 3.84 × 10^-2^ and OR = 2.62, 95 % CI: 2.00-3.43, p = 3.18 × 10^-12^, respectively). The NRI was increased: 4.82% (95% CI: 2.36-7.28, p <0.001), as was IDI, at 0.023 (95% CI: 0.016-0.030, p <0.001). AUC was slightly improved (Δ = 0.011; p = 0.18). | | GRS is associated with IHD (Gui L. et al. 2014) | | |
| 2015 | | | | IHD | | | Prospective cohort analysis | | | Rotterdam population in the Netherlands | | | | Rotterdam Study | | | | 5899 (485 IHD cases at study initiation, 964 additional cases [of which 460 were MI cases] occurred within 12.8 years) | | | | 152 (GRSgws based on 49 genome-wide significant SNPs; GRSfdr using the 103 SNPs that were found in a false discovery rate analysis; GRSall using all 152 SNPs) associated with IHD in CARDIoGRAMplusC4D | | | | Cox proportional hazards model [1) adjustment for sex and age only, 2) adjustment for TRFs, 3) additionally adjusted for family history of MI]. C-statistic, NRI | | | All three GRSs after adjustment for sex and age are associated with IHD, OR: 1.13 (95% CI: 1.06-1.20, p = 0.00014), 1.09 (95% CI: 1.03-1.17, p = 0.0051), and 1.15 (95% CI: 1.08-1.23, p = 1.1 × 10^-5^) for GRS(gws), GRS(fdr), and GRS(all), respectively. After adjustment for TRFs and a family history, OR was weakened, but the association persisted. The greatest improvement of discrimination but not reclassification was obtained for GRS(all) (ΔC = 0.006, 95% CI: 0.000-0.013). GRSs are better associated with IHD at early stages, with improved discrimination [greatest for GRS(all): ΔC = 0.009, 95% CI: 0.003-0.015] and reclassification [except GRS(fdr)]. | | GRSs are associated with IHD but did not result in clinically significant improvements in assessment of 10-year risk of IHD (de Vries P.S. et al. 2015) | | |
| 2015 | | | | IHD | | | Retrospective cohort analysis | | | Population of Southern Sweden | | | | Malmö Diet and Cancer Study (MDCS) | | | | 48,421 individuals and 3477 events | | | | 27 associated with IHD | | | | Cox proportional hazards model adjusted for TRFs | | | Multi-adjusted HR for a high-risk versus low-risk cohort without IHD: 1.72 (95% CI: 1.53-1.92, p < 0.0001) and for recurrent IHD: 1.81 (95% CI: 1.22-2.67, p = 0.0029). Statin therapy reduces the risk of IHD in people at genetic risk. The number needed to treat for IHD depends on the patients’ genetic risk category. | | GRS is associated with IHD, and statin therapy reduces the risk of IHD (Mega J.L. et al. 2015) | | |
|  |  |  |  |  |  |  |  |  |  |  | | | | JUPITER и ASCOT, CARE и PROVE IT-TIMI 22 | | | |  |  |  |  |  |  |  |  |  |  |  |  |  |  |  |  |
| 2015 | | | Recurrent vascular events after ACS (mortality, ACS relapse, second hospitalization due to cardiovascular events within 1 year) | | | Prospective cohort analysis | | | Canada population | | | | Recurrence and Inflammation in the Acute Coronary Syndromes (RISCA) | | | | 1040 (82: death of any causes, ACS relapse, second hospitalization within 1 year) | | | | 30 previously associated with IHD and MI | | | | | Cox proportional hazards model [1) GRS; 2) correction for sex and age; 3) correction for TRFs], IDI and cNRI | | | GRS was not significantly associated with recurrent events (HR = 0.97, 95% CI: 0.91-1.03: RISCA; HR = 0.99, 95% CI: 0.93-1.05: PRAXY; HR = 0.98, 95% CI 0.94-1.02: TRIUMPH; and HR = 0.98, 95% CI: 0.95-1.01: pooled analysis). Only the GRACE scale allowed to predict recurring events in three cohorts (pooled analysis: HR = 0.7, 95% CI: 1.05-1.09, p < 0.001). Adding GRS to the GRACE scale in all cohorts reduces the association, as is the case for IDI and NRI. | | GRS is not associated with an increased risk of recurrent vascular events (Labos C. et al. 2015) | | |
|  |  |  |  |  |  |  |  |  | Populations of Canada, USA, and Switzerland (of European descent) | | | | Premature Acute Coronary Syndrome in men and women (PRAXY) | | | | 691 (93: death of any causes, ACS relapse, second hospitalization within 1 year) | | | |  |  |  |  |  |  |  |  |  |  |  |  |  |
|  |  |  |  |  |  |  |  |  | Individuals of European descent | | | | Translational Research Investigating Underlying disparities in acute Myocardial infarction Patients' Health status (TRIUMPH) | | | | 1772 (214: death of any causes, ACS relapse, second hospitalization within 1 year) | | | |  |  |  |  |  |  |  |  |  |  |  |  |  |
| 2015 | | | MI or IHD | | | Prospective cohort analysis | | | Denmark population | | | | Inter99 | | | | 6041 (IHD: 374 events, MI: 124 events during 11.6 years) | | | | 45 associated with MI, IHD in Europeans according to GWAS | | | | | Cox proportional hazards model [1) correction for age and sex; 2) correction for TRFs of IHD + T2DM]. C-index, IDI, NRI. | | | GRS was associated with MI (model 1: HR = 1.05, 95% CI: 1.01-1.10, p = 0.02; model 2: HR = 1.06, 95% CI: 1.02-1.11, p = 0.01) but was not significantly associated with IHD. The MI risk was more pronounced in older middle-aged individuals (HR = 1.06, 95% CI: 1.00-1.12, p = 0.03) and in males (HR = 1.06, 95% CI: 1.01-1.12, p = 0.02). The addition of MI-associated GRS to the European SCORE algorithm did not improve the NRI and IDI. | | GRS is associated with MI but not with IHD (Krarup N.T. et al. 2015) | | |
| 2015 | | | MI | | | Prospective cohort analysis and case-control | | | Great Britain population | | | | Second Northwick Park Heart Study (NPHSII) | | | | 2775 (284 cases during 13.5 years) | | | | 19 at the loci identified by GWAS; 13 at the loci identified by the CARDIoGRAMplusC4D consortium | | | | | Logistic regression analysis | | | Increased risk of IHD for the higher quintile compared to the lower quintile (for 19 SNPs: p = 18 × 10^-3^, for 13 SNPs: p = 0.01). For the two risk calculators, the NRI was better for 19 SNPs: 4.5% (-3.7-12.7%), p = 0.28, and for 13 SNPs: 3.0% (-4.3-10.3%), p = 0.42, but no statistically significant differences were obtained. The predictive power was comparable to that of the Framingham scale. There were no statistically significant differences in ROC for either 19 SNPs (p = 0.48) or 13 SNPs (p = 0.82). For samples from Pakistan, there was no association between scale quintiles and MI. | | GRSs are associated with MI in the European population but not in the Asian population (Beaney K.E. et al. 2015) | | |
|  |  |  |  |  |  |  |  |  | Pakistan population (Islamabad/ Rawalpindi) | | | | - | | | | 321 cases, 228 controls | | | |  |  |  |  |  |  |  |  |  |  |  |  |  |
|  |  |  |  |  |  |  |  |  | Pakistan population (Lahore) | | | | - | | | | 414 cases, 219 controls | | | |  |  |  |  |  |  |  |  |  |  |  |  |  |
| 2015 | | CVDs (death of cardiovascular causes, nonfatal MI, nonfatal stroke or hospitalization due to angina pectoris) | | | Prospective cohort analysis | | | non-Hispanic whites, African Americans, Spaniards, Native Americans | | | | Look AHEAD (Action for Health in Diabetes) | | | | 4016 patients with T2DM | | | | 153 associated with CVDs | | | | | Cox proportional hazards model [1) lifestyle-adjusted GRS; 2) interaction between GRS and lifestyle] | | Association of GRS with a combined endpoint (death of cardiovascular causes, nonfatal MI, nonfatal stroke, or hospitalization for angina pectoris) (HR: 1.19; 95% CI 1.10, 1.28) and among individuals without a CVD history (HR: 1.18; 95% CI 1.07, 1.30). Lifestyle changes did not affect the association. | | | GRSs were associated with CVDs in all populations (McCafery et al. 2015) | | | |
| 2015 | | CVDs (mortality) | | | Prospective cohort analysis | | | white Americans | | | | DHS (Diabetes Heart Study) | | | | 983 patients with T2DM (all-cause mortality during 10 ± 3 years [mean ± SD] was 31.1%) | | | | 375 taken from the NHGRI GWAS in populations of European origin | | | | | Cox proportional hazards model | | Associated with a coronary artery calcification level (HR = 0.021, 95% CI: 0.010-0.031, p = 1.23 × 10^-4^); however, there was no significant association with MI, cardiovascular events, and all-cause and CVD-caused mortality. | | | GRS is not associated with CVDs (Raffield L.M. et al. 2015) | | | |

**Abbreviations:** ACS: acute coronary syndrome; ARIC: Atherosclerosis Risk in Communities; ATP III: age, systolic blood pressure, hypertensive medication use, smoking, diabetes, and total and high-density lipoprotein cholesterol; AUC: area under the ROC curve; CI: confidence interval; cGRS: counted GRS; CVD: cardiovascular disease; FDR: false discovery ratio; GPS: Genetic Predisposition Score; GRS: Genetic Risk Score; GWAS: genome-wide association study; HDL-C: high-density lipoprotein cholesterol; HR: hazard ratio; IDI: integrated discrimination improvement; IHD: ischemic heart disease; IS: ischemic stroke; LDL-C: low-density lipoprotein cholesterol; MI: myocardial infarction; NRI: net reclassification improvement; OR: odds ratio; Reynolds: age, systolic blood pressure, smoking, diabetes, total and high-density lipoprotein cholesterol, C-reactive protein, and a family history of premature myocardial infarction; RF; risk factor; ROC: receiver-operating characteristic; SBP: systolic blood pressure; SNP: single-nucleotide polymorphism; T2DM: type 2 diabetes mellitus; TC: total cholesterol; TG: triglyceride; TRF: traditional risk factor; wGRS: weighted GRS; UAP: unstable angina pectoris
